# *ARID1A* Regulates Transcription and the Epigenetic Landscape via POLE and DMAP1 While *ARID1A* Deficiency or Pharmacological Inhibition Sensitizes Germ Cell Tumor Cells to ATR Inhibition

**DOI:** 10.3390/cancers12040905

**Published:** 2020-04-07

**Authors:** Lukas Kurz, Alissa Miklyaeva, Margaretha A. Skowron, Nina Overbeck, Gereon Poschmann, Teresa Becker, Katharina Eul, Thomas Kurz, Stefan Schönberger, Gabriele Calaminus, Kai Stühler, Emily Dykhuizen, Peter Albers, Daniel Nettersheim

**Affiliations:** 1 Department of Urology, Urological Research Lab, Translational UroOncology, University Hospital Düsseldorf, 40225 Düsseldorf, Germany; 2 Molecular Proteomics Laboratory, Heinrich-Heine-University Düsseldorf, 40225 Düsseldorf, Germany; 3 Institute for Molecular Medicine I, Medical Faculty, Heinrich-Heine-University Düsseldorf, 40225 Düsseldorf, Germany; 4 Institute for Pharmaceutical and Medicinal Chemistry, Heinrich Heine University Düsseldorf, 40225 Düsseldorf, Germany; 5 Department of Paediatric Haematology and Oncology, University Hospital Bonn, 53113 Bonn, Germany; 6 Department of Medicinal Chemistry and Molecular Pharmacology, Purdue University, West Lafayette, IN 479078, USA; 7 Department of Urology, University Hospital Düsseldorf, 40225 Düsseldorf, Germany

**Keywords:** ARID1A, SWI/SNF-complex, ATR inhibition, HDAC inhibition, germ cell tumors, molecular therapy, CRISPR/Cas9, mass spectrometry

## Abstract

Germ cell tumors (GCTs) are the most common solid malignancies found in young men. Although they generally have high cure rates, metastases, resistance to cisplatin-based therapy, and late toxicities still represent a lethal threat, arguing for the need of new therapeutic options. In a previous study, we identified downregulation of the chromatin-remodeling SWI/SNF complex member ARID1A as a key event in the mode of action of the histone deacetylase inhibitor romidepsin. Additionally, the loss-of-function mutations re-sensitize different tumor types to various drugs, like EZH2-, PARP-, HDAC-, HSP90- or ATR-inhibitors. Thus, ARID1A presents as a promising target for synthetic lethality and combination therapy. In this study, we deciphered the molecular function of ARID1A and screened for the potential of two pharmacological ARID1A inhibitors as a new therapeutic strategy to treat GCTs. By CRISPR/Cas9, we generated *ARID1A*-deficient GCT cells and demonstrate by mass spectrometry that *ARID1A* is putatively involved in regulating transcription, DNA repair and the epigenetic landscape via DNA Polymerase POLE and the DNA methyltransferase 1-associated protein DMAP1. Additionally, *ARID1A/ARID1A* deficiency or pharmacological inhibition increased the efficacy of romidepsin and considerably sensitized GCT cells, including cisplatin-resistant subclones, towards ATR inhibition. Thus, targeting ARID1A in combination with romidepsin and ATR inhibitors presents as a new putative option to treat GCTs.

## 1. Introduction

Testicular type II germ cell tumors (GCTs) are classified into seminomas and non-seminomas. Both entities are thought to arise from a precursor lesion, the so called germ cell neoplasia in situ (GCNIS), which itself is the result of a defective (primordial) germ cell development [1,2]. GCTs represent the most common solid malignancies of young men of age 17–45 years and incidence rates are rising steadily, especially in Western countries [1,3]. In general, GCTs are treated by orchiectomy followed by chemo- or radiotherapy. Despite high cure rates exceeding 90% over all stages, about 20% of patients with advanced disease relapse and become treatment refractory [4,5,6]. Such patients are usually incurable and face a poor prognosis with a life expectancy of a few months only [4,5,6].

In previous studies, we demonstrated the efficacy of the histone deacetylase inhibitor (HDACi) romidepsin in treating (cisplatin-resistant) GCTs [7,8]. Romidepsin induced cell cycle arrest and apoptosis at low nanomolar concentrations (>2 nM) in GCT cells *in vitro* and *in vivo*, but not non-cancerous control cells [7,8].

We identified *ARID1A* downregulation as a key event in the molecular mode of action of romidepsin. Downregulation of *ARID1A*, as a result of histone-hypoacetylation around the promotor, led to upregulation of stress-, apoptosis-, and cell cycle-related genes, like *GADD45B*, *ATF3*, *CDKN1A* and *DUSP1* [7]. ARID1A is a member of the ATP-dependent SWI/SNF chromatin remodeling complex, which plays an important role in cellular senescence, apoptosis and oncogenesis [9]. ARID1A is required for transcriptional activation or repression of genes [9]. Additionally, ARID1A facilitated the DNA damage response of the SWI/SNF-complex and suppression of ARID1A in H1299 and U2OS cells led to reduced non-homologous end joining repair of DNA double strand breaks. Moreover, it was reported that the loss of SMARCA4, another member of the SWI/SNF complex, led to diminished binding of DNA topoisomerase 2-alpha (TOP2A) to DNA in mouse embryonic stem cells [10,11]. This effect was also shown for *ARID1A* mutant HCT116 cells, indicating that the SWI/SNF complex is important for adequate localization of TOP2A [10,11]. Thus, downregulation of *ARID1A* after romidepsin application might also result in an altered transcription rate, DNA synthesis, and DNA damage response. Interestingly, the *ARID1A* gene is mutated (loss-of-function) in a broad spectrum of human malignancies, like ovarian, gastric, breast or bladder tumors [11,12,13,14,15,16,17]. These *ARID1A-*mutated tumors often become sensitive towards other inhibitors, like PARP-, ATR-, EZH2-, HSP90- and HDAC6-inhibtors [11,12,15,16]. Thus, in patients with gynecological cancers, the ATARI trial (NCT04065269) currently studies the response of *ARID1A* deficient subtypes to PARP- and ATR-inhibitors.

In this study, we asked if a romidepsin-mediated downregulation or pharmacological inhibition of ARID1A phenocopies the molecular effects of the *ARID1A* loss-of-function mutation and re-sensitizes GCTs to PARP-, ATR-, EZH2-, HSP90-, and HDAC6-inhibition or cisplatin. Furthermore, we deciphered the molecular consequences of an *ARID1A* deficiency in seminoma-like TCam-2 cells.

## 2. Results

### 2.1. Genomic and Molecular Characterization of ARID1A and the SWI/SNF Complex

**Figure 1:**
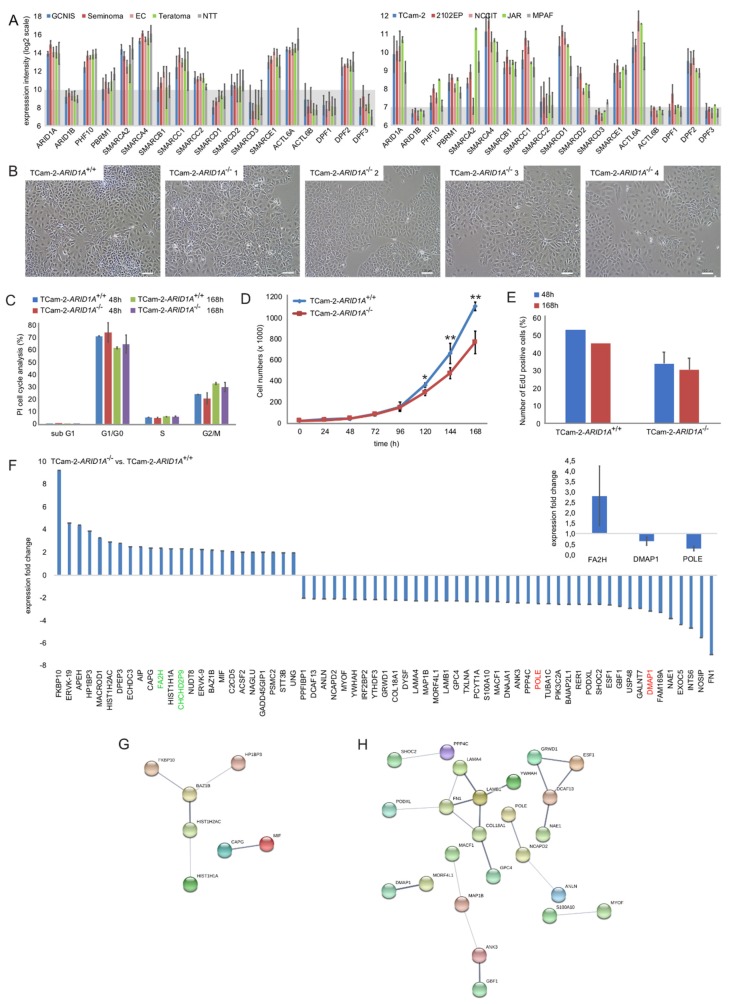
(**A**) Expression microarray data of SWI/SNF complex members in GCT tissues (left) and cell lines (right). Normal testis tissue (NTT) and fibroblasts (MPAF) were included as controls. Data were re-analyzed in context of this study. See ‘materials and method’-section for more details on expression microarray data. (**B**) Brightfield pictures of TCam-2-*ARID1A*^+/+^ and -*ARID1A*^−/−^ cell morphology. Scale bar: 100 μm. (**C**) Measurement of cell cycle phase distribution by PI-based flow cytometry in TCam-2-*ARID1A*^+/+^ and -*ARID1A*^−/−^ cells. (**D**) Growth curves of TCam-2-*ARID1A*^+/+^ and -*ARID1A*^−/−^ clones (average) over seven days. *p*-values * < 0.05, ** < 0.005. (**E**) EdU proliferation assay of TCam-2-*ARID1A*^+/+^ and -*ARID1A*^−/−^ clones (average) after 48 and 168 h. (**F**) Mass spectrometry data of proteins enriched or depleted in TCam-2-*ARID1A*^−/−^ versus -*ARID1A*^+/+^ cells. Inlay: A qRT-PCR analysis confirmed upregulation of *FA2H* and downregulation of *DMAP1* and *POLE*. *GAPDH* was used as housekeeper and for data normalization. (**G**,**H**) STRING-based interaction prediction of enriched (**G**) or depleted (**H**) proteins in TCam-2-*ARID1A*^−/−^ versus -*ARID1A*^+/+^ cells.

The *ARID1A* gene can be transcribed into nine isoforms, four of which are expressed with variable intensities in GCT and testis tissues (Figure S1A, blue, green, yellow, light blue). Only the isoform *EST00000324856.11* encodes for the full length ARID1A protein (Figure S1A, blue). We analyzed the expression of *ARID1A* in various cancers (including GCTs) by screening microarray data of GCT tissues and cell lines as well as the ‘The Cancer Genome Atlas’ (TCGA) pan-cancer dataset (Figure 1A, Figure S1B). *ARID1A* expression was detected in type II GCT tissues (GCNIS, seminomas, ECs, teratomas) and cell lines (TCam-2 (seminoma), 2102EP, NCCIT (ECs), JAR (choriocarcinoma)), while *ARID1B* expression was expressed considerably weaker (Figure 1A). Compared to other common cancer types, GCTs show high *ARID1A* expression (7th place of the 37 analyzed cancer types) (Figure S1B). *ARID1A* expression was also detectable in pediatric type I GCTs (immature and mature teratoma, yolk-sac tumors) (Figure S1B).

Next, we analyzed the DNA methylation profile of *ARID1A* in GCTs. We stratified the TCGA testicular cancer cohort into seminomas (SOX17+) and non-seminomas (SOX2+ (EC), AFP+ (yolk-sac tumor), beta-hCG+ (choriocarcinomas) (Figure S1D). We found a short hypermethylated area embedded in a strongly hypomethylated promotor region and increasing DNA methylation levels towards the 3′-UTR of the *ARID1A* gene locus (Figure S1D). The *ARID1A* DNA methylation profile of GCT cell lines mimicked the profile found in the TCGA pan-cancer cohort (Figure S1D,E). Interestingly, there seems to be a considerable number of seminoma cases that in contrast to non-seminomas show intermediate to low DNA methylation of the region embedded in the promotor and at the 3′ end (Figure S1D). To date, the functional consequence of this finding remains elusive.

We extended our analysis to the expression of SWI/SNF complex members in GCT tissues and cells lines (Figure 1A). As controls, normal testis tissue (NTT) and MPAF fibroblasts were included, respectively. The expression profile was highly similar between GCT tissues and cell lines, with *ARID1A*, *PHF10*, *PBRM1*, *SMARCA2/A4/B1/C1/E1*, *ACTL6A* and *DPF2* being expressed predominantly (Figure 1A). Thus, these factors represent the core members of the SWI/SNF complex in GCTs. Of note, in contrast to tissues, GCT cell lines showed a strong expression of *SMARCB1* and *SMARCD1* (Figure 1A).

We further screened for the mutational burden of these SWI/SNF core members in GCT patients (Figure S1F). Amplifications, deletions or truncations of these genes were extremely rare in GCTs (one truncation in *ARID1A*, one truncation in *PBRM1*, four deletions in *SMARCA2*, n_total_ = 140 patients). Instead, frequently increased mRNA levels of many SWI/SNF members were found (mainly in non-seminomas) (Figure S1F).

### 2.2. Deciphering the Molecular Function of ARID1A in GCTs

To analyze the molecular function of ARID1A in GCTs, we used the CRISPR/Cas9 gene editing technique to generate TCam-2 cells deficient for *ARID1A* (TCam-2-*ARID1A*^−/−^) (Figure S2A–D). Three guideRNAs were transfected to delete a fragment of 2920 bp from the *ARID1A* genomic locus (Figure S2A). GuideRNA-A and -B target exon 2 (guideRNA-A and -B target the same locus, but on opposite strands), while guideRNA-C targets exon 4 (Figure S2A). A PCR strategy using primers flanking guideRNA-A and -C was used to validate an efficient knock out (if successful, the PCR fragment for a homozygous knock out is around 201 bp in size; the ‘wild-type’ fragment is 3121 bp). We successfully generated eight TCam-2-ARID1A^−/−^ clones (Figure S2A). We verified deletion of the 2920 bp fragment in three clones by Sanger sequencing (Figure S2B). Additionally, we confirmed strong reduction/loss of *ARID1A/*ARID1A expression in four clones by qRT-PCR and western blotting (Figure S2C–E). For qRT-PCR, we used primers flanking the different guide RNAs and primers upstream of guideRNA target sites (Figure S2C,D). For western blotting, we used a monoclonal ARID1A antibody (Figure S2E). These data show that no functional *ARID1A* full-length transcript and protein is being produced. These four clones were included in all subsequent analyses. The TCam-2-*ARID1A*^−/−^ cells did not differ from the parental cells (TCam-2-*ARID1A*^+/+^) with regard to cell morphology or cell cycle phase distribution (Figure 1B,C), but showed a reduced proliferation rate over 168 h (Figure 1D,E).

Of note, we were not able to generate *ARID1A*-deficient EC cells (2102EP or NCCIT). In both cell lines, only heterozygous *ARID1A*-knock out clones could be generated, suggesting that a complete loss of *ARID1A* is not tolerated in EC cells (Figure S3A).

To decipher the molecular function of ARID1A in GCTs, we performed mass spectrometry analysis of TCam-2-*ARID1A*^−/−^ and -*ARID1A*^+/+^ cells (Table S1A). We found 24 proteins significantly enriched (fold change (FC) ≥ 2; Anova *p*-value ≤ 0.5) and 42 depleted in TCam-2-*ARID1A*^−/−^ (n = 5) versus -*ARID1A*^+/+^ (n = 5) cells (Figure 1F; Table S1B). As expected, the lowest amount of ARID1A protein was found in TCam-2-*ARID1A*^−/−^ cells (Table S1A). By qRT-PCR analysis, we confirmed upregulation of *FA2H* and downregulation of *DMAP1* and *POLE* (Figure 1F, inlay on right side). By the STRING algorithm, we identified putative networks of the enriched- or depleted proteins (Figure 1G,H). Additionally, we performed DAVID-mediated GeneOntology (GO, GO-MF, GO-BP), KEGG and UniProt (UP) analyses to identify the underlying molecular pathways (Table S1C). On the one hand, the proteins enriched in TCam-2-*ARID1A*^−/−^ versus -*ARID1A*^+/+^ cells (so negatively regulated by ARID1A) were linked to the terms ‘Acetylation’, ‘Protein-binding’, ‘DNA-binding’, ‘ATP-binding’, ‘Nucleotide-binding’, ‘metal-binding’ and ‘zinc finger-binding’ (and others) (Table S1C). On the other hand, proteins depleted in *ARID1A*-deficient TCam-2 (so positively regulated by ARID1A), were linked to ‘Actin-binding’, ‘Cell adhesion’, ‘Cadherin-binding’, Extracellular matrix organization’, ‘Calcium-ion-binding’, ‘Transcription’, ‘Transcription DNA-templated’ and ‘Transcription regulation’ (Table S1C). *ARID1A’s* role in regulating proteins involved in ‘Transcription’, ‘Acetylation’ and ‘DNA/nucleotide binding’ seems quite plausible in light of ARID1A’s bivalent role in the chromatin-remodeling SWI/SNF-complex, i.e., guiding and binding the complex to DNA and regulating gene expression positively and negatively.

### 2.3. Targeting ARID1A as a Therapeutic Option

**Figure 2:**
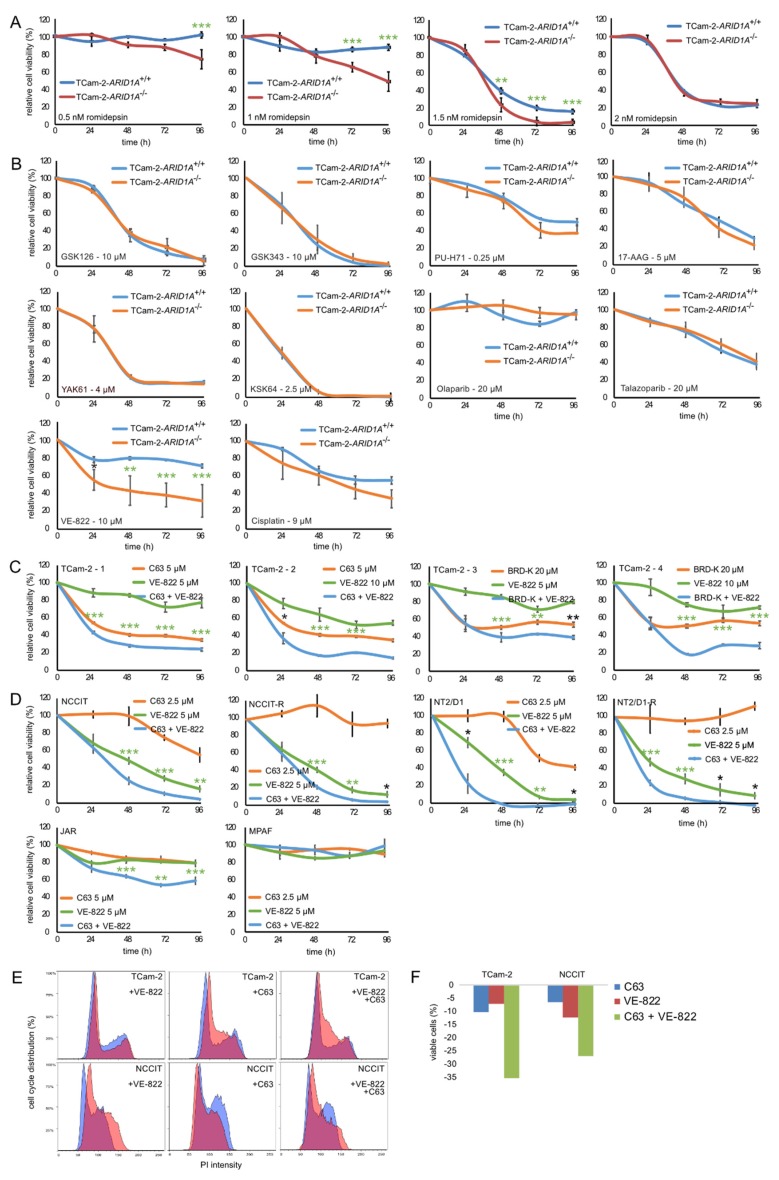
(**A**) XTT data of TCam-2-*ARID1A*^+/+^ and -*ARID1A*^−/−^ clones (average) treated once with 0.5–2 nM romidepsin. (**B**) XTT data of TCam-2-*ARID1A*^+/+^ and -*ARID1A*^−/−^ clones (n = 4) treated once with EZHi GSK126 or GSK343 (both 10 μM), PARPi olaparib or talazoparib (both 20 μM), HSP90i PU-H71 (0.25 μM) or 17-AAG (5 μM), HDAC6i YAK61 (4 μM) or KSK64 (2.5 μM), cisplatin (9 μM) and ATRi VE-822 (10 μM). (**C)** XTT data of TCam-2-*ARID1A*^+/+^ cells treated with C63 (5 μM) or BRD-K (20 μM) alone or in combination with VE-822 (5 μM, 10 μM). (**D**) XTT data of NCCIT(-R), NT2/D1(-R) and JAR cells treated with C63 and/or VE-822. As control, MPAF fibroblasts were included. A–D) Changes in viability (compared to solvent treated controls) were measured over 96 h. *p*-values * < 0.05, ** < 0.005, *** < 0.0005; *p*-values labelled in green were still significant after correction for multiple testing. (**E**,**F**) PI- and Annexin V-based flow cytometry analysis of cell cycle phase distribution (**E**) and apoptosis rates (**F**) in TCam-2 and NCCIT cells treated with C63 and/or VE-822 for 24 h.

Next, we asked, whether ARID1A presents as a promising target for a GCT therapy. In a previous publication, we demonstrated that romidepsin kills GCT cells at a concentration ≥2 nM and identified ARID1A as a key factor in romidepsin’s mode of action [8]. Thus, we tested, whether the efficacy of romidepsin is further boosted in TCam-2-*ARID1A*^−/−^ cells. Indeed, the efficacy of romidepsin was significantly increased in TCam-2-*ARID1A*^−/−^ cells in a small window of 0.5 (>96 h)–1.5 nM (>48 h), while 2 nM romidepsin quickly (<48 h) killed both, TCam-2-*ARID1A*^−/−^ and -*ARID1A*^+/+^ cells (Figure 2A). Thus, at a romidepsin concentration range of 0.5–1.5 nM, *ARID1A* deficiency slightly boots a romidepsin treatment, while at romidepsin concentrations >2 nM, the effects of *ARID1A* deficiency remained neglectable (Figure 2A).

*ARID1A* loss-of-function mutations sensitize cancer cells towards inhibition of EZH2, ATR, PARP, HDAC6, and HSP90 (and others) [11,12,15,16]. Therefore, we asked, whether *ARID1A*-deficiency phenocopies these effects and if TCam-2-*ARID1A*^−/−^ cells become sensitive towards EZH2-, ATR-, PARP-, HSP90- and HDAC6-inhibition as well as to cisplatin application (Figure 2B). First, we narrowed down the optimal treatment concentrations by treating TCam-2-*ARID1A*^+/+^ with various inhibitors, followed by measurement of changes in viability by XTT assays (Figure S3B). Next, we also treated TCam-2-*ARID1A*^−/−^ cells with the optimal inhibitor concentrations.

*ARID1A*-deficiency had no influence on the effect of EZH- (10 μM GSK126; 10 μM GSK343), PARP- (olaparib, 20 μM; talazoparib, 20 μM), HSP90- (0.25 μM PU-H71; 5 μM 17-AAG) and HDAC6-inhibition (0.4 μM YAK61; 2.5 μM KSK64 [18]) or cisplatin (9 μM) on the viability of TCam-2 cells (Figure 2B). In contrast, sensitivity towards ATR-inhibitor VE-822 (10 μM) significantly increased in TCam-2-*ARID1A*^−/−^ cells compared to parental TCam-2 cells (Figure 2B). Subsequently, we asked if this effect can be mimicked by using pharmacological ARID1A inhibitors. Therefore, we treated TCam-2 cells with the ARID1A inhibitors compound 63 (C63; 2.5 μM) and BRD-K98645985 (BRD-K; 20 μM) in combination with the ATR inhibitor VE-822 (5 and 10 μM) (ARID1A inhibitors were applied 24h before VE-822) (Figure 2C) [19,20]. We found an additive effect on viability after ARID1A and ATR inhibition (LD_50_: +VE-822 14.8 μM; +C63 5.7 μM; +VE-822 and C63 0.4 μM) (Figure S2C, S3C).

Of note, treatment of TCam-2 cells with 2.5 μM C63 or 10 μM VE-822 24 h before addition of 1 nM romidepsin reduced viability compared to mono-application only slightly (Figure S3D,E), while combining cisplatin with ATR inhibition strongly reduced viability of TCam-2 cells compared to mono-application (Figure S3F). Thus, combining cisplatin with ATR inhibition might also be a promising approach to treat GCTs.

We extended our analysis to the EC cell lines NCCIT and NT2/D1 as well as their cisplatin-resistant subclones (NCCIT-R, NT2/D1-R) and the choriocarcinoma cell line JAR (+2.5 μM C63 and 5 μM VE-822) (Figure 2D). We confirmed results found in TCam-2, i.e., an additive effect on viability by combined treatment with C63 and VE-822 (Figure 2D). In contrast, in MPAF fibroblast control cells, neither C63 nor VE-822 had a considerable effect on the viability (Figure 2D). To narrow down the effects of C63 and VE-822 on viability in more detail, we analyzed cell cycle distribution and apoptosis levels in TCam-2 and NCCIT cells by staining with PI or AnnexinV/PI for subsequent flow cytometry (Figure 2E,F). We found that mono-application of VE-822 and C63 slightly reduced number of cells in G2/M-phase and viable cells, while the effect was strongly enhanced in a double application of C63 and VE-822 (Figure 2E,F). In conclusion, the pharmacological inhibition of ARID1A by C63 or BRD-K opens a therapeutic window for treatment of GCTs in combination with ATR inhibitors.

### 2.4. Deciphering the Molecular Function of the ARID1A-Inhibitor C63

**Figure 3:**
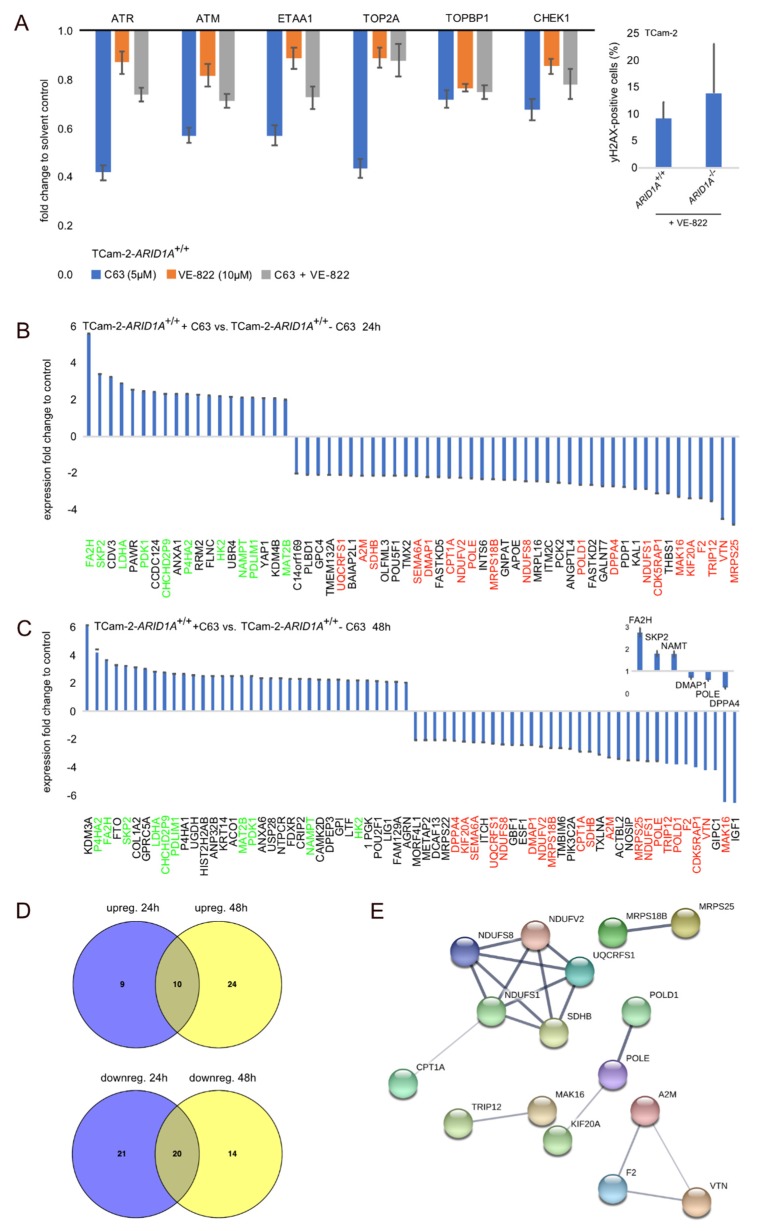
(**A**) qRT-PCR analysis of expression of ATR and DNA damage response associated factors in C63 (5 μM) and VE-822 (10 μM) treated TCam-2 cells. Inlay: Number of phospho-γH2AX-positive cells after 10 μM VE-822 treatment of TCam-2-*ARID1A*^+/+^ and -*ARID1A^−/−^* cells. (**B**,**C**) Mass spectrometry data of proteins enriched or depleted in TCam-2 cells treated for 24 (B) and 48 h (**C**) with C63. Inlay: A qRT-PCR analysis confirmed upregulation of *FA2H, SKP2* and *NAMT* as well as downregulation of *DMAP1*, *POLE* and *DPPA4*. GAPDH was used as housekeeper and for data normalization. (**D**) Venn diagram summarizing commonly enriched or depleted proteins in TCam-2 cells treated for 24 and 48 h with C63. (**E**) STRING-based interaction prediction of proteins commonly depleted in C63 treated TCam-2 (24 + 48 h).

In addition to demonstrating the therapeutic applicability of C63 and BRD-K, we analyzed if the inhibition of ARID1A and ATR affects the expression of the DNA damage response associated factors *ATR*, *ATM*, *ETAA1*, *TOP2A*, *TOPBP1,* and *CHEK1.* These factors play an important role in the impaired DNA damage response related to the ATR pathway in connection to SWI/SNF complex deficiencies [21,22]. All related factors were downregulated in C63, VE-822 or C63 + VE-822 treated TCam-2 cells (Figure 3A). Additionally, in TCam-2-*ARID1A*^−/−^ compared to -*ARID1A*^+/+^ cells, treatment with VE-822 led to an increased number of γH2AX-positive cells (not significant), indicative of DNA double-strand-breaks (Figure 3A, right side; Figure S3G). Thus, inhibition or deficiency of *ARID1A* in combination with ATR inhibition might influence the cellular DNA repair machinery.

We decided to decipher the molecular mode of action of C63 in more detail. Thus, we analyzed changes in the proteome 24 and 48 h after C63 application in TCam-2 cells by mass spectrometry (Table S1A,D,E). After 24 h, we detected 19 proteins significantly enriched (FC ≥ 2; Anova *p*-value ≤ 0.5) and 41 depleted (Figure 3B). After 48 h, we detected 34 proteins enriched and 34 depleted in C63-treated TCam-2 (Figure 3C). Ten proteins were commonly enriched (green labelled; Figure 3B,C) and 20 commonly depleted (red labelled; Figure 3B,C) in 24 and 48 h C63-treated TCam-2 cells (Figure 3D). Again, the STRING algorithm was used to predict interactions. While no interaction was predicted between the ten commonly enriched proteins, a network for the depleted proteins was predicted (Figure 3E). Here, we also used DAVID to screen for correlation to GO-, KEGG-, and UP-categories (Table S1F). Similar to the *ARID1A*-deficient cells, the proteins enriched after C63 application could be linked to ‘Protein-binding’, ‘Metal-binding’, ‘Acetylation’ (among others) (Table S1F). The depleted proteins were linked to ‘NADH activity’, ‘DNA repair’, and ‘Transferase activity’ (Table S1F). Thus, the molecular effects provoked by long-term *ARID1A*-deficiency and short-term inhibition are in parts comparable. Nevertheless, the deregulated proteins were quite different. Only two proteins were found to be commonly enriched (*FA2H*, *CHCHD2P9*) or depleted (*POLE*, *DMAP1*) between *ARID1A*-deficent and C63-treated cells (Figure 1F, red and green labelled; Table S1A). *FA2H* is a fatty-acid hydroxylase, *CHCHD2P9* is a Coiled-Coil-Helix-Coiled-Coil-Helix Domain Containing 2 Pseudogene 9, *POLE* is DNA Polymerase Epsilon and *DMAP1* is a DNA Methyltransferase 1 associated protein.

### 2.5. The Effect of ARID1A-Deficiency on the Pluripotency Program

Due to the involvement of ARID1A in regulating the pluripotency program, we analyzed the effect of *ARID1A*-deficiency or -inhibition on the expression of pluripotency factors [7,23,24,25,26]. Interestingly, long-term cultivated TCam-2-*ARID1A*^−/−^, 2102EP-*ARID1A*^+/-^ and NCCIT-*ARID1A*^+/−^ cells showed no considerable deregulation of *NANOG*, *OCT3/4* or *SOX2/SOX17* (Figure S4A). Although we found downregulation of pluripotency factors in short-term C63- or BRD-K-treated TCam-2, 2102EP or NCCIT cells (Figure S4B). In JAR cells, no expression of pluripotency factors was detectable (Figure S4B). Thus, in TCam-2, downregulation of pluripotency factors is restricted to short-term *ARID1A*-interference (si/shRNA [7] or inhibition), but compensated in long-term *ARID1A*-knock out cells. Further, we speculate that we were not able to generate homozygous *ARID1A*-deleted EC cells, because these cells lose pluripotency, subsequently leading to induction of differentiation and thus cannot be kept in culture for multiple passages.

## 3. Discussion

### 3.1. ARID1A Deficiency or Inhibition Sensitizes GCT Cells to ATR Inhibition

**Figure 4:**
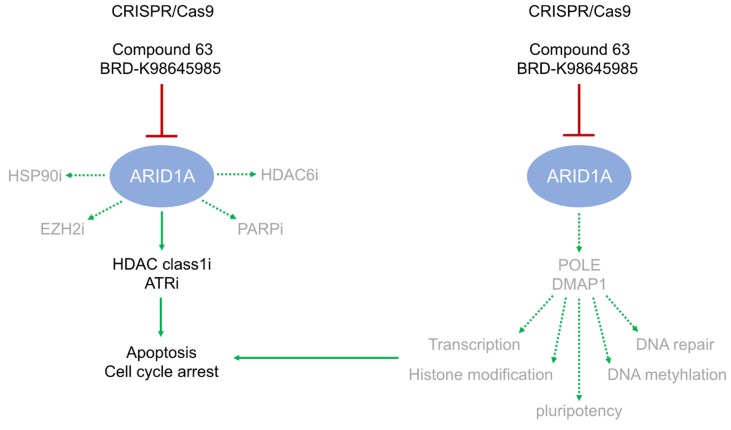
Illustration of the effects of ARID1A inhibition and molecular functions of ARID1A. Deficiency or pharmacological inhibition of ARID1A sensitizes GCTs cells slightly to HDAC class 1 inhibitor romidepsin and more prominently to ATR inhibition, causing a reduction in viability, apoptosis, and cell cycle arrest (left side). On a molecular level, ARID1A normally regulates transcription, DNA repair, DNA methylation, histone modifications and the pluripotency program at least in parts via POLE and DMAP1 (right side). These regulatory cascades diminish upon inhibition of ARID1A, subsequently contributing to the induction of apoptosis and cell cycle arrest. Arrow: sensitization/activation; dotted arrow: no sensitization/diminished activation; T-shaped arrow: inhibition/inactivation.

In this study, we analyzed the molecular role of ARID1A and its suitability as a therapeutic target in the treatment of GCTs. In a previous study, we identified downregulation of *ARID1A* as a key event in the molecular cascade of the HDAC inhibitor romidepsin, subsequently leading to induction of stress, apoptosis, and cell cycle arrest [7]. Loss-of-function *ARID1A* mutations sensitized various cancer cells towards inhibition of EZH2, PARP, ATR, HSP90 and HDAC9 (and others). In GCTs, *ARID1A* mutations are extremely rare, so we asked, if a romidepsin-mediated *ARID1A* downregulation, a CRISPR/Cas9-mediated *ARID1A* deficiency, or a pharmacological inhibition of ARID1A might phenocopy these effects of the *ARID1A* mutation and sensitize GCT cells to the various previously mentioned inhibitors. While *ARID1A* deficiency had no effect on HSP90, PARP, HDAC6, and EZH2 inhibition, the efficacy of the HDAC inhibitor romidepsin and ATR inhibitor VE-822 was significantly enhanced (Figure 4); an effect also observed upon pharmacological inhibition of ARID1A by C63 and BRD-K (Figure 4). Thus, targeting ARID1A in combination with HDAC class I (i.e., romidepsin) and ATR inhibitors presents as a promising therapeutic approach for *ARID1A* unmutated GCTs.

### 3.2. POLE and DMPA1 are Putative ARID1A Effectors

On a molecular level, in GCTs ARID1A’s function was linked to DNA-based regulation of transcription, cell adhesion, and DNA repair, which reflects the role of ARID1A in the SWI/SNF complex, i.e., guiding the complex to DNA and regulating transcription negatively and positively. We identified *POLE* and *DMAP1* as putative downstream effectors regulated by ARID1A (Figure 4). Both factors are involved in and linked to transcriptional activation and repression, chromatin organization, and DNA damage seem to be positively regulated by *ARID1A* [27,28]. Additionally, DMAP1 interacts with the Polycomb repressive complex 1 (PRC1), DNA methyltransferase 1 (DNMT1) and histone deacetylases, such as HDAC2, especially at sites of double-strand break repair [27,28,29,30]. Thus, DMAP1 is also involved in shaping the epigenetic landscape on DNA and histone level. Thus, we hypothesize that the molecular function of ARID1A as part of the chromatin-remodeling SWI/SNF complex (i.e., regulation of transcription and DNA repair by chromatin-remodeling) is putatively mediated by POLE and DMAP1 (Figure 4). Proteins enriched in expression in *ARID1A*-deficient cells were linked to GO-categories ‘Acetylation’, ‘Chromosome’ and ‘DNA-/nucleotide-/nucleic acid-/metal-/ATP-/protein-binding’, suggesting that related molecular functions are repressed by ARID1A in TCam-2.

### 3.3. ARID1A Transiently Affects the Pluripotency Program in Seminoma and EC Cells

In long-term cultivation, we suggest that *ARID1A*-deficient cells try to counteract the diminished activity of ARID1A by upregulating alternative factors that take over the molecular function of ARID1A. This idea is in line with the finding that many deregulations in gene expression found in short-term *ARID1A*-interfered cells (RNAi; [9])/pharmacological inhibition (C63, BRD-K)) normalized in long-term *ARID1A*-knock-out cells. For example, downregulation of pluripotency factors, like *NANOG*, *OCT3/4* and *DPPA4* was found only in RNAi-mediated *ARID1A* knock-down or C63/BRD-K-mediated ARID1A-inhibited cells [9] (Figure 4; Figure S4). Thus, ARID1A negatively influences expression of pluripotency factors, but in long-term culture seminoma cells (TCam-2) can counteract this effect (Figure 4). Interestingly, EC cells are not able to counteract ARID1A-mediated deregulations of the pluripotency program and induce differentiation instead. This might be the reason, why we were able to generate only heterozygous *ARID1A*-deletions in EC cells and no homozygous deletions. In EC cells, at least 50% of normal *ARID1A* expression needs to be maintained without inducing differentiation.

## 4. Material and Methods

### 4.1. Ethics Statement

The ethics committee of the Heinrich-Heine-University Düsseldorf (EC-HHU-D) raised no concerns about using all analyzed cell lines for *in vitro* experiments and drug screening (ethics votes 2018-178 and 2019-412). All type I GCT samples were cryopreserved and anonymized during the MAKEI 96-study and provided by the MAKEI 96-biobank with no concerns raised by EC-HHU-D about analyzing these samples (ethic votes 837 and 2019-822).

### 4.2. Cell Culture

All GCT cell lines were cultivated as described previously [7,31]. The cisplatin-resistant GCT sublines were kindly provided by Friedemann Honecker (ZeTuP Silberturm, St. Gallen, Switzerland) and Christoph Oing (University Hospital Eppendorf, Hamburg, Germany). MPAF were kindly provided by Dr. Michael Peitz (Life & Brain, Department of Reconstructive Neurobiology, Bonn, Germany). STR profiles of all cell lines are checked on a regular basis and are available upon request.

### 4.3. Inhibitor Preparation and Application

The ARID1A inhibitors compound 63 and BRD-K98645985 (Prof. Dr. Emily Dykhuizen), the EZH2-inhibitor GSK126 (Cayman Chemical, Ann Arbor, MI, USA), the PARP-inhibitors olaparib and rucaparib (Cayman Chemical), the ATR inhibitors VE-822 and AZD6738 (Cayman Chemical), the HSP90 inhibitors PU-H71 and 17-AAG (Selleckchem, Houston, USA), the HDAC6 inhibitors YAK61 and KSK64 (Prof. Dr. Thomas Kurz, [18]) were dissolved in DMSO. The EZH2 inhibitor GSK343 (Cayman Chemical) was dissolved in DMF, while cisplatin (Accord Healthcare, Munich, Germany) was dissolved in a saline solution.

### 4.4. Gene Editing

TCam-2 cells deficient for *ARID1A* were generated by the CRISPR/Cas9 technique as described previously [7,32,33]. Briefly, by using FuGeneHD (Promega, Walldorf, Germany), 1 × 10^5^ TCam-2 cells were transfected simultaneously with 1 μg pX330 vector encoding for three different guideRNAs directed against the *ARID1A* gene locus (Santa Cruz, Heidelberg, Germany) and 100 ng of a GFP-encoding plasmid (pEGFP-N3). Clones were screened for deletions within the *ARID1A* gene locus by PCR. For genotyping strategy, see Figure S2A (Figure S2A). See Table 1 for guideRNA sequences (Table 1).

### 4.5. Measurement of Cell Viability

The XTT assays were performed as described previously [7]. In summary, 4 × 10^3^ cells were plated onto 96-well plates 24 h before inhibitor or solvent application. For in a total of 96 h, every day viability was screened by adding 50 μL XTT (1 mg/mL) plus 0.5 μL phenazine methosulfate (PMS, 1.25 mM) (both from Sigma-Aldrich, Taufkirchen, Germany) and measuring absorbance 4 h later in a UV/VIS spectrometer (450 nm vs. 650 nm, BioRad, Düsseldorf, Germany). Each time point/concentration was measured in quadruplicates.

### 4.6. Flow Cytometry

Flow cytometry analyses of apoptosis rates and cell cycle distribution were performed by AnnexinV/propidium iodide (PI) or PI-staining, respectively. Briefly, for PI staining, cells were washed once with PBS and fixed with 70% methanol dissolved in PBS. After washing, cells were re-suspended in staining buffer, containing 2 μg/mL PI (Sigma-Aldrich) and 100 μg/mL RNAse A (Qiagen, Hilden, Germany) dissolved in PBS before measurement. For apoptosis measurement, cells were washed with Annexin V-binding buffer (Miltenyi, Bergisch Gladbach, Germany) before incubation with 2.5 μL Annexin V-FITC (Miltenyi) and 7.5 μL PI (2 mg/mL stock, Sigma-Aldrich) dissolved in 70 µL Annexin V-binding buffer for 15 min at room temperature (RT) in the dark. Afterwards, samples were re-suspended in 500 µL Annexin V-binding buffer. Measurement of cell proliferation was performed using the EdU Flow Cytometry Kit detecting Eterneon-Red 645 Azide according to the manufacturer’s protocol (Baseclick, Neuried, Germany). A flow cytometric analysis was performed by measuring at least 5 × 10^4^ cells using the MACSQuant Analyser and evaluated by the Flowlogic software (both Miltenyi Biotech).

### 4.7. Immunofluorescence

For immunofluorescent staining (IF), 5 × 10^4^ cells/well were seeded onto 96-well plates. After 24 h, cells were treated with either VE-822 or DMSO as a solvent control for additional 24 h. For staining of phospho-Histone H2A.X Ser139 (Cell Signaling Technology, Frankfurt am Main, Germany), cells were fixed by 4% formaldehyde for 10 min. Afterwards, cells were permeabilized by 0.3% Triton-X in PBS for 10 min and blocked in PBS (+10% goat serum, 0.3 M glycine, and 0.1% Triton-X) for 1 h at RT. The primary antibody (1:50) was incubated in PBS staining solution (+1% BSA, and 0.1% Triton-X) over night at 4 °C. The secondary antibody was added for 1 h in the dark at RT. Cells were counter-stained with 0.5 µg/mL 4′,6-Diamidine-2′-phenylindole dihydrochloride (DAPI, Sigma-Aldrich). See Table 2 for antibody details (Table 2).

### 4.8. Measurement of Cell Growth

Cellular growth rates were determined by counting cell numbers every day over eight days (each in triplicates). Initially, 4 × 10^3^ cells were seeded out.

### 4.9. DNA, RNA and Protein Isolation

For RNA extraction, cells were harvested and RNA was isolated using the RNAeasy Mini Kit (Qiagen, Hilden, Germany) according to the manufacturer’s protocol. For protein extraction, cells were incubated with RIPA buffer containing 10% protease- and phosphatase-inhibitors (Sigma-Aldrich). Protein concentration was assessed by BCA Protein Assay Reagent Kit (Thermo Fisher Scientific, Dreieich, Germany).

### 4.10. Western Blot

An amount of 20 µg of whole protein lysates was used for western blotting. Staining with Ponceau S-solution (Sigma-Aldrich, 0.5% in 5% acetic acid) confirmed a correct membrane transfer. Membranes were blocked in 5% non-fat milk in PBS + 1% Tween20 (PBST) for 1 h and then incubated with primary antibodies (Table 2) over night at 4 °C. Subsequently, secondary HRP-conjugated antibodies were incubated for 2 h at RT. The ChemiDoc Imaging System (BioRad) was used for imaging. See Table 2 for antibody details (Table 2).

### 4.11. Sanger Sequencing

For Sanger sequencing of ARID1A-deficient clones, PCR amplified DNA was cloned into the pCR2.1 vector using the ‘TA cloning kit’ (Thermo Fisher Scientific) and following the manual guidelines (ratio insert DNA to vector: 3:1). For genotyping primers see Table 1 (Table 1). TOP10 *Escherichia coli* bacteria were transformed according to the ‘TA cloning kit’ manual. Plasmid DNA was isolated from bacteria mini-preps using the ZR plasmid miniprep kit (Zymo Research, Freiburg, Germany). An EcoR1-HF restriction enzyme (New England Biolabs, Frankfurt am Main, Germany) double digest of 1 μg plasmid DNA for 30 min at 37 °C, followed by agarose-gel-electrophoresis, confirmed insertion of the PCR product into the plasmid. Plasmids were sequenced by M13 primers at the ‘Genomics & Transcriptomics Laboratory’ (Heinrich-Heine-University, Düsseldorf, Germany).

### 4.12. Mass Spectrometry: Sample Preparation

Proteins were extracted from frozen cell pellets as described elsewhere [34]. Briefly, cells were lysed and homogenized in urea buffer with a TissueLyser (Qiagen) and supernatants were collected after centrifugation for 15 min at 14,000× *g* and 4 °C, supernatants were collected. Protein concentration was determined by means of Pierce 660 nm Protein Assay (Thermo Fischer Scientific) and 10 µg protein per sample were loaded on a SDS-PAGE for in-gel-digestion. The isolated gel pieces were reduced (50 µL, 10 mM DTT), alkylated (50 µL, 50 mM iodoacetamide) and underwent afterwards tryptic digestion (6 µL, 200 ng trypsin in 100 mM ammonium bicaonate). The peptides were resolved in 0.1% trifluoracetic acid and subjected to liquid chromatography.

### 4.13. Mass Spectrometry: LC-MS Analysis

For the LC-MS analysis, a QExactive plus (Thermo Fisher Scientific) connected with an Ultimate 3000 Rapid Separation liquid chromatography system (Dionex/Thermo Fisher Scientific, Idstein, Germany) equipped with an Acclaim PepMap 100 C18 column (75 µm inner diameter, 25 cm length, 2 mm particle size from Thermo Fisher Scientific) was applied. The length of the LC gradient was 120 min. The mass spectrometer was operating in positive mode and coupled with a nano electrospray ionization source. The capillary temperature was set to 250 °C and source voltage to 1.4 kV. In the QExactive plus mass spectrometer for the survey scans, a mass ranging from 200 to 2000 m/z at a resolution of 140,000 was used. The automatic gain control was set to 3,000,000 and the maximum fill time was 50 ms. The ten most intensive peptide ions were isolated and fragmented by high-energy collision dissociation.

### 4.14. Mass Spectrometry: Computational Mass Spectrometric Data Analysis

Proteome Discoverer (version 2.3.0.532, Thermo Fisher Scientific) was applied for peptide/protein identification applying Mascot (version 2.4, Matrix Science, London, UK) as search engine employing the human database (Uniprot, Swissprot). A false discovery rate of 1% on peptide level was set as identification threshold. Proteins were quantified with Progenesis QI for Proteomics (Version 2.0, Nonlinear Dynamics, Waters Corporation, Newcastle upon Tyne, UK). Only proteins containing at least two unique peptides were taken into consideration. For the calculation of enriched proteins in the groups a 5% false discovery rate and a minimum fold change of ≥2 was used.

The mass spectrometry proteomics data were deposited to the ProteomeXchange Consortium via the PRIDE partner repository with the data set identifier PXD017898 [35].

### 4.15. Quantitative RT-PCR

A total amount of 1µg of RNA was in-vitro transcribed into cDNA by using 1 µL dNTP-Mix (10 mM), 1 µL Oligo(dT)18 Primer (0.5 µg/µL), 4 µL RT Buffer (5×), 1 µL Maxima H Minus Reverse Transcriptase (200U/µL), and 0.5 µL RiboLock RNase Inhibitor (40U/µL) (all Thermo Fisher Scientific) on a S1000 cycler (BioRad) at 52 °C for 0.5 h. Further, qRT-PCR runs were performed on a 384-well C1000 cycler (BioRad). In general, all samples were analyzed in technical triplicates using 7.34 ng of cDNA for each replicate and the SYBR-green-based Luna Universal qPCR Master Mix (New England Biolabs, Frankfurt am Main, Germany). At the end of each run, melting curve analyses were performed. Oligonucleotide sequences are given in Table 1 (Table 1).

### 4.16. Illumina HT-12v4/Affymetrix Expression Arrays and Illumina 450k DNA Methylation Array

The Illumina and Affymetrix expression array analyses of GCT cell lines (TCam-2, n = 5; 2102EP, n = 5, NCCIT, n = 4; JAR, n = 2; FS1, n = 4; MPAF, n = 4) and tissues (GCNIS, n = 3; seminomas, n = 4; ECs, n = 3; teratomas, n = 3; normal testis tissue, n = 4) were performed previously and re-analyzed in context of this study [7,8,33,36,37,38,39]. Illumina microarray expression data of parental GCT cell lines is also available via GEO (ncbi.nlm.nih.gov/geo/) (GSE71239, GSE71269, GSE79065, GSE60698). For Affymetrix microarrays, expression intensities of <10 and for Illumina microarray expression intensities of <7 were considered as ‘not expressed’. Thresholds were set based on the expression intensity of *SOX2* and *SOX17* in seminomas (*SOX2*−, *SOX17*+) and ECs (*SOX2*+, *SOX17*−). The Illumina 450k DNA methylation arrays were generated in a previous study and re-analyzed in context of this study (GSE76709) [7].

### 4.17. Online Analyses Tools

Venn diagrams were generated using Venny 2.1 (https://bioinfogp.cnb.csic.es/tools/venny/) [40]. The STRING algorithm was used to predict networks by confidence using default settings (https://string-db.org) [41]. Functional annotation analyses were performed by DAVID (https://david.ncifcrf.gov/home.jsp) [42,43]. For functional annotation clustering by DAVID, only cluster with a Kappa similarity term overlap of 3, a similarity threshold of 0.5, an initial and final group membership of 5, a multiple linkage threshold of 0.5 and an enrichment threshold of 1 were included. TCGA datasets were analyzed using the UCSC Xena browser (https://xena.ucsc.edu), FireBrowse (http://firebrowse.org) and the cBioPortal (https://www.cbioportal.org) [44,45]. Testing for significance was performed via the ‘Social Sciences Statistics T-Test Calculator for 2 Independent Means’ (https://www.socscistatistics.com/tests/studentttest/default.aspx) using a Student’s two-tailed *t*-test.

### 4.18. Statistical Analysis

Where appropriate, two tailed t-tests were calculated with α = 5%. *p*-values of * < 0.05, ** < 0.005 and *** < 0.0005 were assumed as ‘statistically significant’. Additionally, *p*-values were corrected for multiple testing by the Bonferroni method (0.05/number of analyses). *p*-values below the significance threshold after correction are labelled in green.

## 5. Conclusions

In this study, we deciphered the molecular mode of action of the romidepsin-effector and SWI/SNF-complex member ARID1A in GCTs. We demonstrated that ARID1A might be involved in regulating transcription and the epigenetic landscape, putatively via DNA polymerase POLE and the DNMT-, HDAC- and PrC-interacting DMAP1. Furthermore, pharmacological inhibition of ARID1A increased efficacy or romidepsin and sensitized GCT cells to ATR inhibition. In summary, we added new pieces to the puzzle of ARID1A’s complex molecular functions in GCTs and highlighted ARID1A as an interesting target for combinatorial treatment with HDAC class I and ATR inhibitors.

## 6. Limitations of This Study

This study also has some limitations we would like to summarize here.

Regarding our CRISPR/Cas9-based *ARID1A* knock-out: based on the detection of protein fragments smaller than the ARID1A wild-type protein (270 kDa) by western blotting, we cannot rule out leftovers of a truncated ARID1A protein with unknown functionality. Additionally, although the *ARID1A* guideRNAs target exon 2 and 4 off all *ARID1A* isoforms, we cannot fully exclude that remaining fragments of other *ARID1A* isoforms or related proteins, e.g., ARID1B are redundant to ARID1A and might take over its function.

Furthermore, seminoma-like TCam-2 cells might not be an ideal model to study DNA methylation levels of ARID1A in seminomas, since TCam-2 harbor untypically high levels of DNA methylation.

To confirm regulation of DMAP1 and POLE by ARID1A, further functional experiments will be necessary in the future.

The expression microarray data re-analyzed in this study, has been gathered from GCT tissues. Although, all tissues were stained by IHC for related marker genes and checked by a pathologist, we cannot fully exclude that immune cells, i.e., lymphocytes that express *ARID1A*, were present to a certain degree. Although, expression of typical immune cell marker genes was very low in analyzed tissues and expression data of tissues was highly similar to cell lines (Table S1G; Figure 1A).

To confirm that targeting ARID1A and ATR inhibition presents as a therapeutic option, testing of more cell lines of different GCT entities as well as xenograft experiments is mandatory.

## Figures and Tables

**Table 1:** Oligonucleotides used in this study.

Gene	Forward primer	Reverse primer	Tan	Cycles
ARID1A-5″	TCTTGCCCATCTGATCCATT	CCAACAAAGGAGCCACCAC	60 °C	40
ARID1A-flank. guideRNA A,B	CGCAGCAAGGACATGGGTA	ATGGAGTCTGGCCCTGTTGA	60 °C	40
ARID1A-flank. guideRNA C	TCTCAGCAGTCCCAGCAAAC	AGGCAAGCTGGAGGGTCTT	60 °C	40
ARID1B	CAAGGGGATCAGAGCAACCC	CTACCTGGGATACTTGCAGGA	60 °C	45
ATF3	AAGAACGAGAAGCAGCATTTGAT	TTCTGAGCCCGGACAATACAC	60 °C	40
ATM	TGGATCCAGCTATTTGGTTTGA	CCAAGTATGTAACCAACAATAGAAGAAGTAG	60 °C	40
ATR	CAGCTTTGTGCCATTTACTG	CTACCTCAATTCCAAGCACA	60 °C	40
CDKN1A	CCTCATCCCGTGTTCTCCTTT	GTACCACCCAGCGGACAAGT	60 °C	40
CHEK1	ATATGAAGCGTGCCGTAGACT	TGCCTATGTCTGGCTCTATTCTG	60 °C	40
DUSP1	GTACATCAAGTCCATCTGAC	GGTTCTTCTAGGAGTAGACA	60 °C	40
ETAA1	GAGAATTTCCATACATTTCCCCTTT	CTAAACAAGGAAGTAATTTGGTACAATCAA	60 °C	40
FGF4	TTCTTCGGGCCATGAGCAG	CCGAAGAAAGTGCACCAAGG	60 °C	40
FOS	GAGAGCTGGTAGTTAGTAGCATGTTGA	AATTCCAATAATGAACCCAATAGATTAGTTA	60 °C	45
GADD45B	GTCGGCCAAGTTGATGAAT	CACGATGTTGATGTCGTTGT	60 °C	40
GAPDH	TGCCAAATATGATGACATCAAGAA	GGAGTGGGTGTCGCTGTTG	60 °C	45
ID2	TCAGCCTGCATCACCAGAGA	CTGCAAGGACAGGATGCTGATA	60 °C	40
MLH1	CTTGTACCCCCCGGAGAAG	TGCAACATCTCCCGGAGAAC	60 °C	40
P53	TTGCAATAGGTGTGCGTCAGA	AGTGCAGGCCAACTTGTTCAG	60 °C	40
SMARCA4	CAGCATGCCAAGGATTTCAAG	CGATCCGCTCGTTCTCTTTC	60 °C	40
SMARCB1	AACGTCAGCGGGTTCAAAT	GCCTTCACCTGGAACATGAA	60 °C	40
TOP2A	AGTCATTCCACGAATAACCA	TTCACACCATCTTCTTGAG	60 °C	40
TOPBP1	TGTGACTGGCTTATGTGGCT	TGGCACACTCATACTTCTGACC	60 °C	40
TP53BP1	ATTGAGGATACGGAACCCATGT	TGCTGGATTCATCAGGATACTATCA	60 °C	40
XPC	GGCCAAAGGTCTGCTCATCA	GTCCACCTCCTGCATCTGTG	60 °C	40
ZMYND11	TTGTTAAACGTGCCATGACC	GCATGTGTGGAGACAGAGGA	60 °C	40
ARID1A genotyping	GCGGTACCCGATGACCATGC	TACTGGAGGTCATTGAGGGG	60 °C	45
ARID1A guideRNA A	GCGGTACCCGATGACCATGC			
ARID1A guideRNA B	ATGGTCATCGGGTACCGCTG			
ARID1A guideRNA C	CCCCTCAATGACCTCCAGTA			

**Table 2:** Antibodies used in this study.

Antibody	Company	Clone	Order No.	Dilution	Application
primary antibodies					
ARID1A	Cell Signaling Technology	D2A8U	12354	1:500	Western Blot
Beta-Actin	Sigma-Aldrich	AC-15	A5441	1:20,000	Western Blot
GAPDH	Abcam	6C5	ab8245	1:30,000	Western Blot
Vinculin	Merck/Sigma	V284	05–386	1:2000	Western Blot
Phospho-Histone H2A.X	Cell Signaling Technology	Ser139	2577	1:50	IF
secondary Abs					
Polyclonal Rabbit Anti-Mouse HRP	Dako		P026002-2	1:1000	Western Blot
Polyclonal Goat Anti-Rabbit HRP	Dako		P044801-2	1:2000	Western Blot
Goat anti-Mouse IgG (H+L) Alexa Fluor 488	Thermo Fisher Scientific		A11029	1:2000	IF/FACS
Goat anti-Rabbit IgG (H+L) Alexa Fluor 488	Thermo Fisher Scientific		A11034	1:2000	IF/FACS

## Supplementary Materials

The following are available online at https://www.mdpi.com/2072-6694/12/4/905/s1, **Figure S1:** (**A**) Xena browser-mediated analysis of ARID1A ISOFORM expression in GCT and testis tissues. (**B**) FireBrowse-mediated analysis of ARID1A expression in various cancer types. GCTs were framed in red. (**C**) qRT-PCR analysis of *ARID1A* expression in type I GCT tissues and type II GCT cell lines. *GAPDH* was used as housekeeper and for data normalization. (**D**) The TCGA testicular cancer cohort was stratified into seminomas (*SOX17*+ (B)) and non-seminomas (*SOX2*+ ECs (C), *AFP*+ yolk-sac tumors (D) and *beta-hCG*+ choriocarcinomas (E)) and screened for *ARID1A* DNA methylation levels using the Xena browser (F). (**E**) Illumina 450k microarray data of *ARID1A* DNA methylation in the GCT cell lines TCam-2, 2102EP, NCCIT and JAR. Data were re-analyzed from [7,8,33,36,37,38,39] in context of this study. (**F**) cBioPortal-mediated analysis of genetic alterations of SWI/SNF complex members in GCT patients, **Figure S2**: (**A**) Illustration of the CRISPR/Cas9 and genotyping strategy to generate and validate *ARID1A* deficiency in GCT cells (left). A successful generation of homozygous knock out clones was checked by genotyping PCR and visualized by agarose gel electrophoresis (right). (**B**) By Sanger sequencing of three clones, deletion of a 2920 bp fragment within the genomic sequence of *ARID1A* was confirmed. (**C**–**E**) qRT-PCR (**C**), (**D**) and Western blot (**D**) analyses of *ARID1A*/*ARID1A* expression confirms strong downregulation in four different TCam-2-*ARID1A*^−/−^ compared to TCam-2-*ARID1A*^+/+^ cells. *GAPDH* (**C**), (**D**) and VINCULIN (**E**) were used as housekeepers and for data normalization. For qRT-PCR, primers flanking the guideRNA target sites (**C**) and primers upstream of guideRNA target sites (**D**) were used, while for western blotting a monoclonal ARID1A antibody was used (**E**), **Figure S3**: (**A**) Generation of heterozygous *ARID1A*-knock out 2102EP and NCCIT clones was demonstrated by genotyping PCR and visualized by agarose gel electrophoresis (right). (**B**) XTT data of TCam-2-*ARID1A*^+/+^ cells treated once with indicated concentrations of the EZHi GSK126 or GSG323, PARPi olaparib or talazoparib, HSP90i PU-H71 or 17-AAG, HDAC6i YAK61 or KSK64, ATRi VE-822, cisplatin and the ARID1A inhibitors C63 and BRD-K. Changes in viability (compared to solvent treated controls) were measured over 96 h. (**C**) LD_50_ values of C63- or VE-822-treated TCam-2-*ARID1A*^−/−^ and TCam-2-*ARID1A*^+/+^ cells. (**D**–**F**) XTT data of TCam-2-*ARID1A*^+/+^ cells treated once with indicated concentrations of C63 or VE-822 in combination with romidepsin (D, E) or cisplatin (**F**). Changes in viability (compared to solvent treated controls) were measured over 96 h. *p*-values * < 0.05, ** < 0.005, *** < 0.0005; *p*-values labelled in green were corrected for multiple testing and are significant. (**G**) Example of immunofluorescent staining of phospho-γH2AX in a TCam-2-*ARID1A*^−/−^ clone and TCam-2-*ARID1A*^+/+^ cells after 10 μM VE-822 treatment for 24 h. Scale bar: 100 μm, **Figure S4**: (**A**,**B**) qRT-PCR expression analysis of pluripotency factors in TCam-2-*ARID1A*^−/−^, 2102EP-*ARID1A*^+/-^ and NCCIT-*ARID1A*^+/-^ cells (**A**) as well as TCam-2, 2102EP, NCCIT, and JAR cells treated with C63 (10 μM) or BRD-K (10 μM) for 24h (**B**). *GAPDH* and *ACTB* were used as housekeepers and for data normalization, **Table S1**: Detailed analyses of mass spectrometry data.
