# Supplementary material for: ARID1A Regulates Transcription and the Epigenetic Landscape via POLE and DMAP1 While ARID1A Deficiency or Pharmacological Inhibition Sensitizes Germ Cell Tumor Cells to ATR Inhibition
Source: Cancers (Basel). 2020 Apr 7;12(4):905. doi: 10.3390/cancers12040905 (PMC7226530; doi:10.3390/cancers12040905)
Supplement: Supplementary file 1 [file cancers-12-00905-s001.zip › supplementarry (figures).docx]

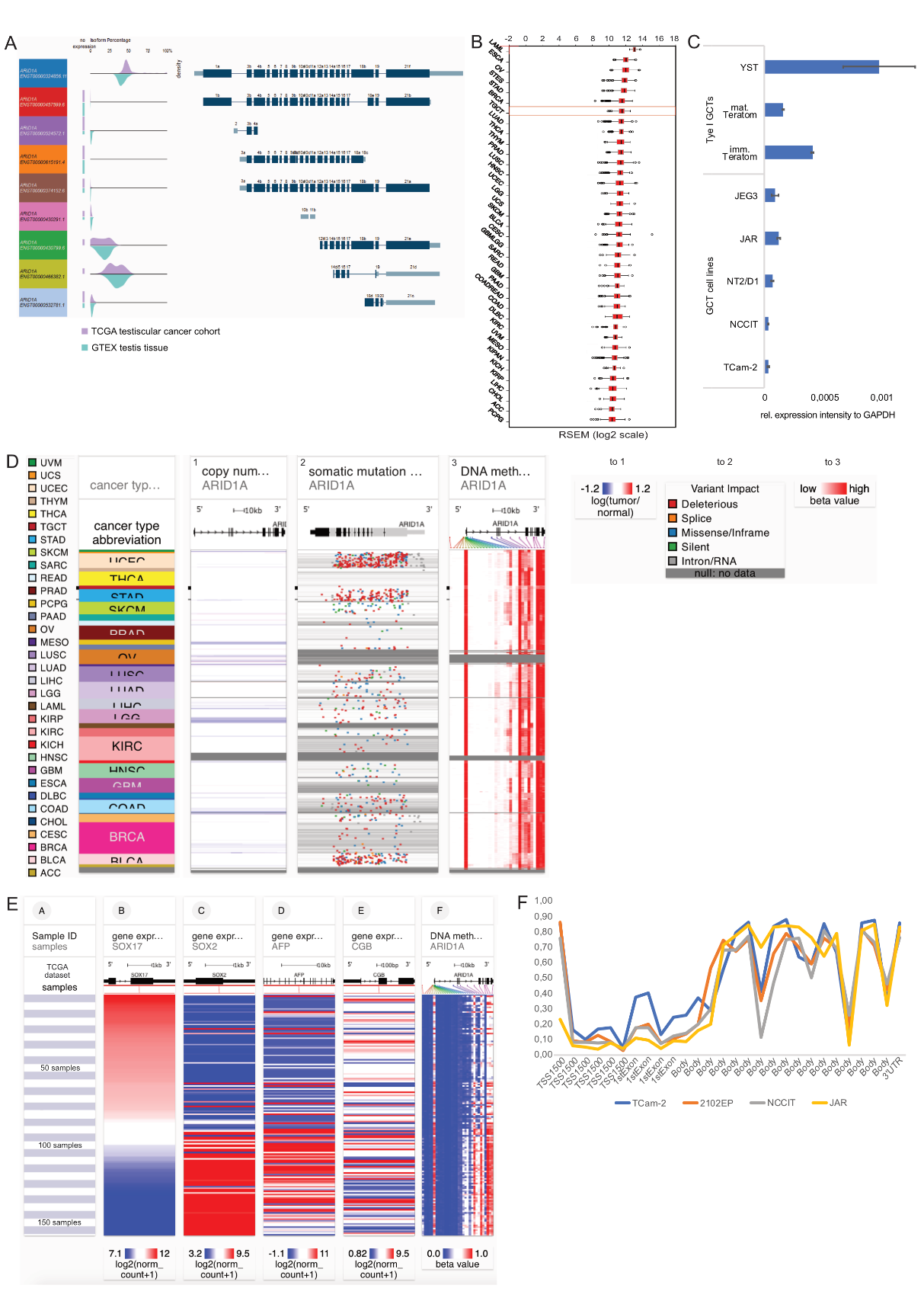


**Figure S1:** (**A**) Xena browser-mediated analysis of ARID1A isoform expression in GCT and testis tissues. (**B**) FireBrowse-mediated analysis of ARID1A expression in various cancer types. GCTs were framed in red. (**C**) qRT-PCR analysis of *ARID1A* expression in type I GCT tissues and type II GCT cell lines. *GAPDH* was used as housekeeper and for data normalization. (**D**) The TCGA testicular cancer cohort was stratified into seminomas (*SOX17*+ (B)) and non-seminomas (*SOX2*+ ECs (C), *AFP*+ yolk-sac tumors (D) and *beta-hCG*+ choriocarcinomas (E)) and screened for *ARID1A* DNA methylation levels using the Xena browser (F). (**E**) Illumina 450k microarray data of *ARID1A* DNA methylation in the GCT cell lines TCam-2, 2102EP, NCCIT and JAR. Data were re-analyzed from [7,8,33,36–39] in context of this study. (**F**) cBioPortal-mediated analysis of genetic alterations of SWI/SNF complex members in GCT patients
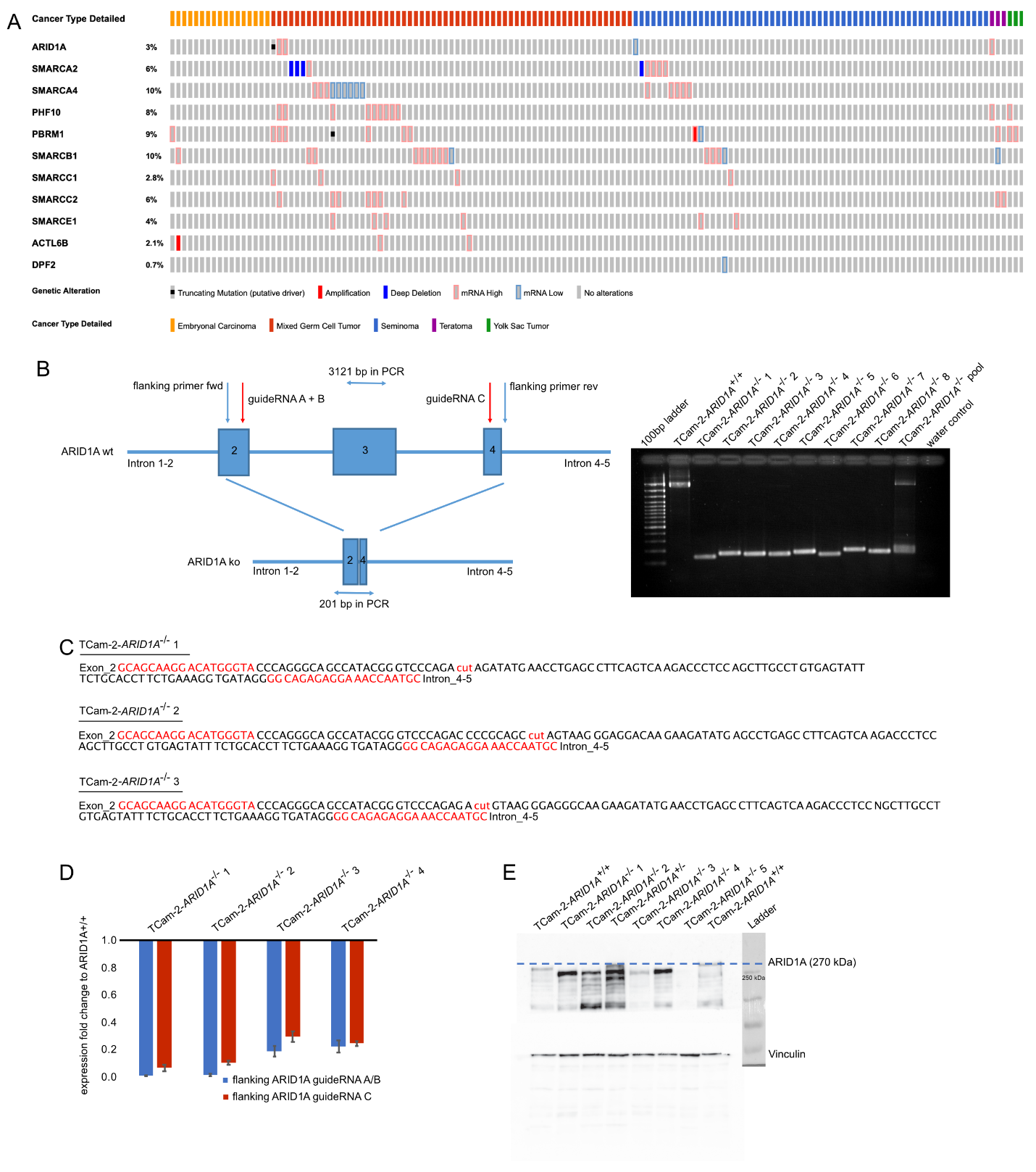


**Figure S2:** A) cBioPortal-mediated analysis of genetic alterations of SWI/SNF complex members in GCT patients. B) Illustration of the CRISPR/Cas9 and genotyping strategy to generate and validate *ARID1A* deficiency in GCT cells (left). A successful generation of homozygous knock out clones was checked by genotyping PCR and visualized by agarose gel electrophoresis (right). C) By Sanger sequencing of three clones, deletion of a 2920 bp fragment within the genomic sequence of *ARID1A* was confirmed. D, E) A qRT-PCR (D) and western blot (E) analysis of *ARID1A* / ARID1A expression confirms strong downregulation in four different TCam-2-*ARID1A*^-/-^ compared to TCam-2-*ARID1A*^+/+^ cells. *GAPDH* (D) and VINCULIN (E) were used as housekeepers and for data normalization. No densitometric evaluation has been performed since band intensities of knock out samples at 270 kDa equal the background and thus are zero.


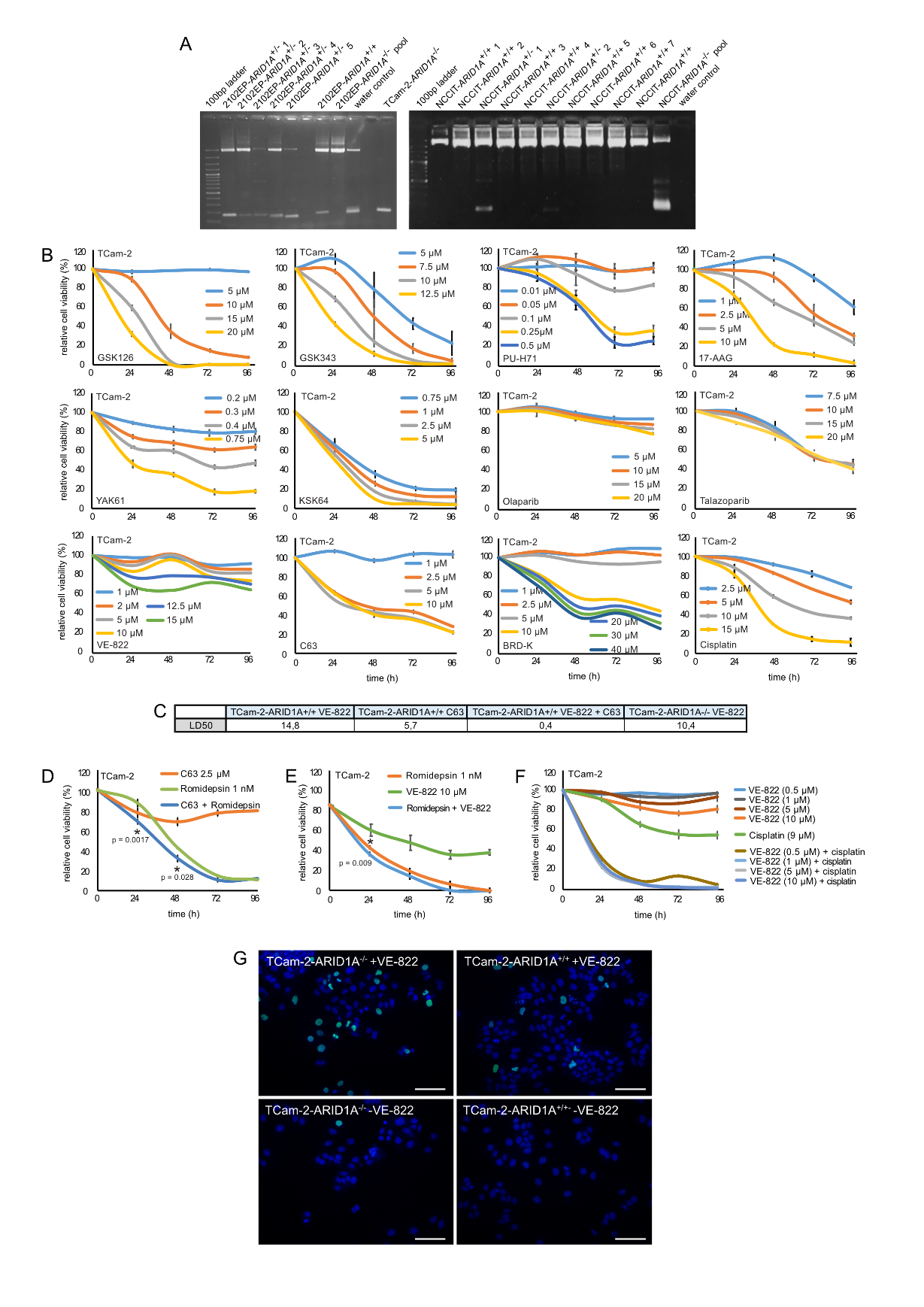


**Figure S3**: (**A**) Generation of heterozygous *ARID1A*-knock out 2102EP and NCCIT clones was demonstrated by genotyping PCR and visualized by agarose gel electrophoresis (right). (**B**) XTT data of TCam-2-*ARID1A*^+/+^ cells treated once with indicated concentrations of the EZHi GSK126 or GSG323, PARPi olaparib or talazoparib, HSP90i PU-H71 or 17-AAG, HDAC6i YAK61 or KSK64, ATRi VE-822, cisplatin and the ARID1A inhibitors C63 and BRD-K. Changes in viability (compared to solvent treated controls) were measured over 96 h. (**C**) LD_50_ values of C63- or VE-822-treated TCam-2-*ARID1A*^-/-^ and TCam-2-*ARID1A*^+/+^ cells. **(D–F)** XTT data of TCam-2-*ARID1A*^+/+^ cells treated once with indicated concentrations of C63 or VE-822 in combination with romidepsin (D, E) or cisplatin (**F**). Changes in viability (compared to solvent treated controls) were measured over 96 h. *p*-values *  0.05, ** < 0.005, *** < 0.0005; *p*-values labelled in green were corrected for multiple testing and are significant. (**G**) Example of immunofluorescent staining of phospho-γH2AX in a TCam-2-*ARID1A*^-/-^ clone and TCam-2-*ARID1A*^+/+^ cells after 10 μM VE-822 treatment for 24 h. Scale bar: 100 μm,
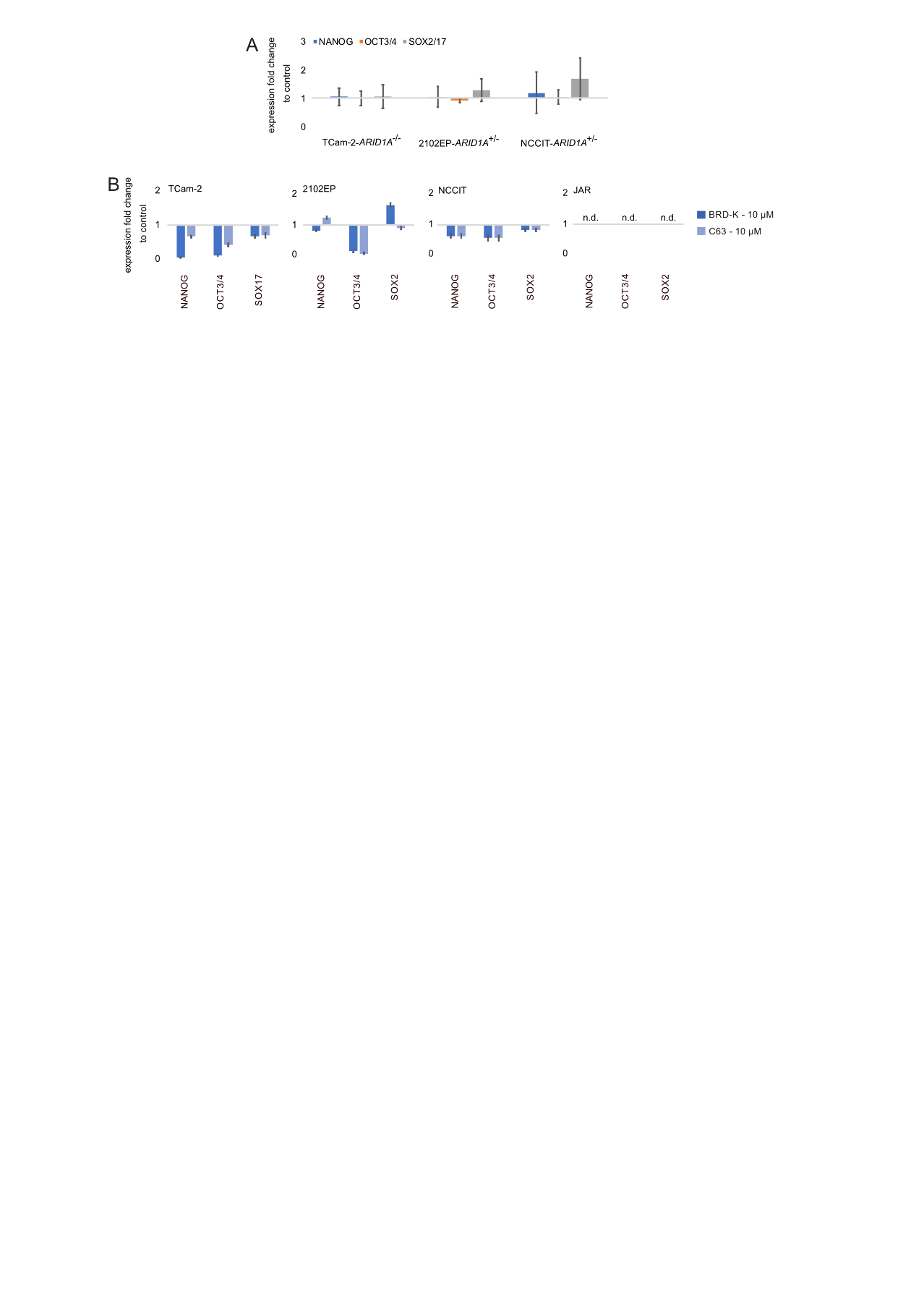


**Figure S4**: (**A**,**B**) qRT-PCR expression analysis of pluripotency factors in TCam-2-*ARID1A*^-/-^, 2102EP-*ARID1A*^+/-^ and NCCIT-*ARID1A*^+/-^ cells (**A**) as well as TCam-2, 2102EP, NCCIT, and JAR cells treated with C63 (10 μM) or BRD-K (10 μM) for 24h (**B**). *GAPDH* and *ACTB* were used as housekeepers and for data normalization, Table S1: Detailed analyses of mass spectrometry data.
